# Dramatic escalation in metabolic syndrome and cardiovascular risk in a Chinese population experiencing rapid economic development

**DOI:** 10.1186/1471-2458-14-983

**Published:** 2014-09-20

**Authors:** Xiang Qian Lao, Wen Jun Ma, Tanja Sobko, Yong Hui Zhang, Yan Jun Xu, Xiao Jun Xu, Dong Mei Yu, Shao Ping Nie, Qiu Mao Cai, Xiao Lin Wei, Liang Xia, Martin Chi-sang Wong

**Affiliations:** School of Public Health and Primary Care, Chinese University of Hong Kong, Hong Kong, Hong Kong; Shenzhen Research Institute of the Chinese University of Hong Kong, Shenzhen, China; Guangdong Provincial Institute of Public Health, Center for Disease Control and Prevention of Guangdong Province, Guangzhou, China; Institute of Human Performance, The University of Hong Kong, Hong Kong, Hong Kong; Center for Disease Control and Prevention of Guangdong Province, 160, Qun Xian Road Pan Yu District, Guangzhou, China; National Institute for Nutrition and Food Safety, Chinese Center for Disease Control and Prevention, Beijing, China

**Keywords:** Metabolic syndrome, Cardiovascular risk, Trend, Economic development, Chinese

## Abstract

**Background:**

Metabolic syndrome (MetSyn) increases the incidence of cardiovascular disease. Information on changes in prevalence of MetSyn in developing countries is limited. This study aims to compare MetSyn prevalence and its associated vascular risk over the period between 2002 and 2010 in a population which has had the world’s fastest economic development over the past three decades.

**Methods:**

Two health surveys were conducted by using the multistage cluster random sampling method in a Chinese population of 85 million in southern China. The participants received a full medical check-up, including measurement of blood pressure (BP), obesity indices, fasting lipids and glucose levels. Data describing socio-economic status and lifestyle factors were also collected through interview. Metabolic syndrome was defined in accordance with the International Diabetes Federation criteria.

**Results:**

A total of 3,561 participants from Survey 2010 were included in the data analysis. Women had a significantly higher prevalence of MetSyn than men. Comparison between the two surveys shows that age-standardized prevalence of MetSyn increased fourfold (from 5.4% in 2002 to 21.3% in 2010) in those ≧ 20 years. Among the MetSyn components, prevalence of hyperglycaemia has increased most (from 9.1% to 53.1%). The age-standardized prevalence of central obesity, hypertension, hypertriglyceridaemia and low HDL-cholesterol increased from 13.5% to 25.4%, from 23.6% to 40.8%, from 12.1% to 17.4% and from 32.1% to 71.1%, respectively. Differences between rural and urban residents in the prevalence in MetSyn and its components narrowed in 2010.

**Conclusions:**

Cardiovascular risk escalated dramatically in this population between 2002 and 2010. The escalation may relate to the rapid economic development, which led to accelerating changes in nutrition, lifestyle, and socio-economic status. Our findings suggest that health transition in rapidly developing second- and third-world countries may be much faster than what has been observed in Western countries.

## Background

Cardiovascular disease is the leading cause of mortality both in China and worldwide [1, 2]. A constellation of cardiovascular risk factors consisting of abdominal obesity, hypertension, elevated fasting glucose level and dislipidemia defines the metabolic syndrome (MetSyn) [3]. It is estimated that around 38% of adults in the U.S.A. [4], and at least 25% of the adult in Europe suffer from MetSyn [5–7]. A MetSyn epidemic is also emerging in certain developing countries in Asia, Latin America, and parts of Africa [8–10], where it has become a serious public health problem. The emerging MetSyn epidemic in developing countries is thought to be related to rapid industrialization and urbanization [11]. However, information on the development of the epidemic and the growth rate of MetSyn in developing countries experiencing rapid economic development is limited.

China is the world’s largest developing country and along with rapid economic growth and urbanization it is experiencing an epidemic of cardiovascular disease. Previous studies have shown that MetSyn is generally prevalent in the Chinese population. However, the degree of prevalence varies across the different regions of China because of marked differences in demographic characteristics, cultural behavior, and lifestyle habits [12–16]. Furthermore, despite the fastest GDP in the world in the past three decades, there is little information on the growth rate of MetSyn in Chinese. Guangdong is a province in southern China with a population of 85 million [17]. During the past three decades, Guangdong experienced an average annual increase of 13.6% in GDP, the largest of all China’s 34 provinces and autonomous regions [18]. Guangdong therefore provides a unique opportunity to observe changes in MetSyn prevalence representing a rapid epidemiological transition resulting from industrialization and urbanization. Results we previously published show that MetSyn was already prevalent in 2002 [19]. To elucidate the present situation, and to observe changes in MetSyn as well as individual risk factors in this rapidly developing society, we compared data from the 2002 and 2010 editions of the Guangdong Health Survey. Such comparisons can be valuable for physicians and policy-makers charged with developing prevention and intervention programs for populations undergoing rapid economic development and urbanization.

## Methods

Approvals were obtained from the Ethics Committee of the China Center for Disease Control, and the Ethics Committee of the Guangdong Provincial Center for Disease Control and Prevention. A total of four standardized Health Surveys were conducted between 2002 and 2010 [20]. The prevalence of MetSyn in 2002 has been previously reported [19]. No blood samples were collected in 2004 and 2007, so it was not possible to calculate the prevalence of MetSyn for these two years. Data for the 2010 survey were collected from October to December. Blood samples were collected from a subsample of 3600 residents; details of the sampling method have been described elsewhere [21].

A central survey site was set up in each selected cluster. Health examinations and interviews of participants were conducted on-site following standard protocols by physicians who received training specifically for the 2010 survey. All participants gave written informed consent prior to the survey. The questionnaire collected a wide range of information about demographic, lifestyle and family characteristics as well as personal disease histories.

Weight and height measurements were taken with participants wearing light indoor clothing and no shoes. All these measures were taken in the morning before breakfast. Waist circumference was measured horizontally around the narrowest circumference between the ribs and the iliac crest. Body mass index (BMI) was defined as weight in kilograms divided by height in square meters.

BP measurement was in accordance with the 1999 World Health Organization/International Society of Hypertension guidelines on hypertension [22]. Participants were required to rest for at least five minutes in a seated position before measurement. Three BP measurements, one minute apart, were taken on the right arm with the participant seated. The average of the last two readings was used for analysis.

Venous blood samples were drawn in the morning after an overnight fast using vacutainer tubes. Fasting plasma glucose (FPG) was measured by a spectrophotometer 721/722 with the glucose oxidase method. Total cholesterol, triglyceride and HDL-cholesterol were determined using a Hitachi 7060 Automatic Chemical Analyzer at the CDC laboratory. Methodology of the above blood sample measurements are the same as the survey in 2002 [18].

The International Diabetes Federation (IDF) criteria were used to define MetSyn because this definition takes account of ethnic difference in waist circumference (also called central obesity) [23]. According to the IDF criteria, participants are classified as having MetSyn if they have central obesity (waist circumference of ≧ 90 cm for men and of ≧ 80 cm for women) plus any other two abnormalities from the four shown immediately below:

1. Hypertension: systolic blood pressure ≧ 130 mmHg, or diastolic blood pressure ≧ 85 mmHg, or treatment of previously diagnosed hypertension;
2. Hypertriglyceridemia: ≧ 1.7 mmol/l of triglyceride or specific medical treatment for this lipid abnormality;
3. Hypo-HDL-cholesterol: < 1.03 mmol/l of HDL-cholesterol for men or of < 1.29 mmol/l for women; and
4. Raised fasting glucose: overnight ≧ 5.6 mmol/l of plasma glucose, or previously diagnosed diabetes.

To allow comparison with the prevalence of MetSyn in 2002 [19], we included for analysis only participants aged ≧ 20 years in the present analysis. This left us with 3,561 participants. All data analyses were performed using SAS software, version 9.2 (SAS Institute, Cary, NC, U.S.A.). As in our previous studies [19–21, 24, 25], we incorporated the survey design parameters including weighting, stratum and cluster, into all the analyses. The weighting was derived from provincial 2000 census data and associated administrative data. Age-standardized prevalence was calculated based on the year 2000 census population by using age groups of 20–39 years, 40–59 years, and 60– years. Data were presented separately for comparison in different sex and urban/rural area. Urban and rural area can be used as a proxy for socio-economic status in China due to the sharp divide between urban and rural areas in income, health care, school quality, access to public goods such as housing, sanitation, and other dimensions of welfare [26, 27]. The classification of urban and rural areas was defined in the early 1990s by Chinese central government based on their level of economic development of the time [28]. Two sided *p* values of less than 0.05 were considered to be statistically significant. 95% confidence intervals were calculated and presented in the present study.

## Results

The mean age of the population aged 20 years or above was 50.4 (42.6, 58.1) years. Urban men had a lower smoking prevalence than rural men, whereas rural women had a higher prevalence than urban women. Men had higher education levels, more tobacco and alcohol consumption, higher systolic BP, diastolic BP and triglyceride levels, and lower HDL-cholesterol levels than women. The anthropometric, blood pressure and plasma biochemical characteristics of the population stratified by sex and urban/rural residence are presented in Table 1.

**Table 1:** **Anthropometric, blood pressure and plasma biochemical characteristics in the population aged 20 years or above by sex and urban/rural residence in 2010**

		Men (1,604)	Women(1,957)	*p*
Age (years)	Urban	52.3 (43.9, 60.6)	50.7 (43.4, 60.6)	0.024
	Rural	50.4 (36.4, 64.4)	49.2 (39.0, 59.5)	0.28
	*p*	0.66	0.65	
Body mass index (kg/m^2^)	Urban	23.4 (21.3, 25.6)	23.3 (21.4, 25.2)	0.31
	Rural	22.9 (22.8, 22.9)	22.5 (21.2, 23.8)	0.34
	*p*	0.36	0.28	
Waist circumference (cm)	Urban	82.6 (75.4, 89.7)	78.0 (74.8, 81.3)	0.019
	Rural	81.6 (77.6, 85.7)	78.2 (66.8, 89.7)	0.14
	*p*	0.68	0.95	
Systolic blood pressure (mmHg)	Urban	135 (133, 138)	128 (127, 129)	0.0025
	Rural	133 (122, 144)	128 (119 137)	0.0015
	*p*	0.46	0.96	
Diastolic blood pressure (mmHg)	Urban	82 (79, 86)	80 (79, 80)	0.024
	Rural	81 (78, 85)	79 (77, 80)	0.009
	*p*	0.63	0.51	
Total cholesterol (mmol/L)	Urban	4.25 (4.01, 4.49)	4.50 (4.09, 4.50)	0.11
	Rural	4.16 (4.07, 4.24)	4.17 (4.02, 4.32)	0.84
	*p*	0.72	0.41	
HDL-cholesterol (mmol/L)	Urban	0.97 (0.77, 1.17)	1.15 (1.01, 1.28)	0.014
	Rural	0.99 (0.95, 1.02)	1.08 (1.02, 1.13)	< 0.001
	*p*	0.83	0.44	
Triglyceride (mmol/L)	Urban	1.48 (1.29, 1.67)	1.19 (0.74, 1.64)	0.030
	Rural	1.45 (1.20, 1.71)	1.16 (1.00, 1.32)	0.056
	*p*	0.85	0.86	
Fasting glucose (mmol/L)	Urban	6.41 (5.55, 7.27)	6.27 (5.72, 6.81)	0.16
	Rural	5.75 (5.04, 6.47)	5.78 (4.66, 6.90)	0.79
	*p*	0.085	0.19	
Education (less than primary school education, %)	Urban	11.0 (0.0, 23.7)	19.0 (0.0, 40.9)	< 0.001
	Rural	9.0 (0.0, 18.5)	30.7 (26.1, 35.3)	< 0.001
	*p*	0.59	0.058	

Values were presented in mean (95% CI) for continuous variables and prevalence (95% CI) for categorical variables.

The overall prevalence of MetSyn in this population was 24.5%. When compared the differences between urban and rural residents, there were no significant differences in the prevalence of MetSyn as well as the individual components except for hypertriglyceridaemia. Men had a lower prevalence of MetSyn and central obesity, but a higher prevalence of hypertension and hypertriglyceridaemia and Hyperglycaemia than women. The prevalence of MetSyn and its components stratified by sex and urban/rural residence are presented in Table 2.

**Table 2:** **Prevalence (95% CI) of individual components of the metabolic syndrome based on the International Diabetes Federation guidelines in southern Chinese aged 20 years or above by sex and urban/rural residence in 2010**

		Men (%)	Women (%)	*p*
Central obesity	Urban	21.3 (3.8, 38.9)	38.2 (20.7, 55.7)	< 0.001
	Rural	17.1 (2.0, 32.1)	38.9 (0.0, 87.9)	< 0.001
	*p*	0.42	0.95	
Hypertension	Urban	61.0 (52.6, 69.3)	46.5 (44.4, 48.6)	< 0.001
	Rural	57.9 (49.7, 66.1)	45.2 (36.0, 54.4)	< 0.001
	*p*	0.27	0.55	
Hypertriglyceridaemia	Urban	27.5 (19.9, 35.1)	20.4 (13.5, 27.3)	< 0.001
	Rural	22.4 (16.3, 28.4)	13.8 (9.4, 18.1)	< 0.001
	*p*	0.023	<0.001	
Low HDL-cholesterol	Urban	64.0 (40.4, 87.7)	69.0 (61.9, 76.1)	0.17
	Rural	62.5 (55.4, 69.5)	79.2 (78.0, 80.4)	< 0.001
	*p*	0.79	< 0.001	
Hyperglycaemia	Urban	76.2 (57.0, 95.4)	70.8 (58.4, 83.1)	0.009
	Rural	47.7 (4.7, 90.8)	42.3 (2.8, 81.8)	< 0.001
	*p*	0.0077	0.0029	
MetSyn	Urban	19.8 (3.2, 36.3)	33.0 (16.6, 49.5)	< 0.001
	Rural	14.1 (2.5, 25.7)	29.3 (0.0, 63.7)	< 0.001
	*p*	0.22	0.68	
MetSyn for all residents by sex		16.3 (6.8, 25.8)	30.9 (9.7, 52.0)	< 0.001

The prevalence of MetSyn increased significantly with age. The prevalence of MetSyn in the population stratified by age group, sex and urban/rural residenceare presented in Table 3.

**Table 3:** **Prevalence (%, 95% CI) for metabolic syndrome among adults aged 20 years or above by age, sex and urban/rural residence in southern China in 2010**

		Total (n = 3,561)	Age (years)			*p* for trend
			20–39 (n = 812)	40–59 (n = 1,911)	60– (n = 838)	
All regions						
	Men	16.3 (6.8, 25.8)	12.0 (0.0, 26.0)	17.0 (12.7, 21.2)	18.5 (2.8, 34.2)	<0.001
	Women	30.9 (9.7, 52.0)	20.4 (5.0, 35.8)	32.6 (12.3, 52.8)	38.0 (12.6, 63.4)	<0.001
	Total	24.5 (8.9, 40.1)	16.9 (2.9, 30.9)	25.9 (13.9, 37.9)	28.6 (9.0, 48.2)	<0.001
Urban						
	Men	19.8 (3.2, 36.3)	18.3 (0.0, 41.6)	19.8 (9.4, 30.2)	20.7 (0.0, 44.7)	< 0.001
	Women	33.0 (16.6, 49.5)	20.7 (10.7, 30.7)	33.7 (13.4, 54.0)	42.9 (0.0, 92.5)	0.099
	Sub-total	27.5 (11.2, 43.8)	19.7 (15.9, 23.5)	28.4 (12.6, 44.2)	31.9 (0.0, 67.7)	0.094
Rural						
	Men	14.1 (2.5, 25.7)	8.5 (0.0, 18.0)	15.2 (14.8, 15.6)	16.9 (0.0, 40.4)	< 0.001
	Women	29.3 (0.0, 63.7)	20.3 (0.0, 43.4)	31.6 (0.0, 64.8)	34.7 (2.3, 67.02)	< 0.001
	Sub-total	22.5 (0.0, 46.2)	15.4 (0.0, 34.6)	24.2 (6.4, 41.9)	26.2 (0.1, 52.4)	< 0.001

The age-standardized prevalence of MetSyn increased fourfold (from 5.4% in 2002 to 21.3% in 2010). Among the components, the greatest increase was for hyperglycaemia, from 9.1% to 53.1%, but dramatic increases were also found for central obesity, hypertension, low HDL-cholesterol and hypertriglyceridaemia. The comparison between 2002 and 2010 of the age-standardized prevalence rates of MetSyn and its components are presented in Table 4.

**Table 4:** **Comparison between 2002 and 2010 of the age-standardized prevalence (%, 95% CI) for the metabolic syndrome among adults 20 years of age or above in southern China**

	Survey 2002	Survey 2010
Central obesity	13.5 (10.6, 16.5)	25.4 (18.1, 32.7)
Hypertension	23.6 (20.9, 26.4)	40.8 (38.7, 42.9)
Hypertriglyceridaemia	12.1 (9.4, 14.8)	17.4 (12.6, 22.2)
Low HDL-cholesterol	32.1 (27.4, 36.8)	71.1 (65.1, 77.1)
Hyperglycaemia	9.1 (6.9, 11.3)	53.1 (38.4, 67.8)
MetSyn	5.4 (4.3, 6.5)	21.3 (15.1, 24.5)

## Discussion

Comparisons of the prevalence of MetSyn across populations are difficult because of differences in the definition of MetSyn by international authorities. Many organizations have tried to develop a unifying definition since Reaven raised the term “syndrome X” in 1988. The definitions frequently used in research include the criteria proposed by World Health Organization (WHO), the European Group for the Study of Insulin Resistance (EGIR), the US National Cholesterol Education Program Adult Treatment Panel III (NCEP ATP III), the American Association of Clinical Endocrinologists (AACE), the International Diabetes Federation (IDF) and the American Heart Association/National Heart, Lung, and Blood Institute (AHA/NHLBI). The general principles in each definition are similar but the cutoffs and thresholds for the variables are somewhat different [29]. So far, the most common used definitions in China are NCEP ATP III and IDF criteria. Previous studies show that the prevalence of MetSyn in China is already high. In an InterASIA study conducted by Gu *et al*. in 2005 [12], the age-standardized prevalence of MetSyn, defined by the modified National Cholesterol Education Programme Adult Treatment Panel III (NCEP ATP III), in a nationwide representative sample of adults between 35 and 74 years of age in China was 9 · 8% for men and 17 · 8% for women. Yang *et al*. found that the prevalence of MetSyn in China was higher according to the NCEP ATP III definition of MetSyn than according to the IDF definition (23.3% vs 16.5%) [30]. In addition, MetSyn prevalence was generally higher in northern China than in southern China. A study in adult Americans aged 20 years or above by Ford *et al*. using the IDF definition found the prevalence of the MetSyn was 34.5% [31], which is higher than that in our population. Brazil and India are the two Gold Brick Four Countries, which are considered as having similar rapid economic development to China. Under the NCEP ATP III definition, the prevalence of MetSyn in India ranged from 11.0% to 32.5% [32], while in Brazil, Marquezine reported a prevalence of 25.4% [10].

Although it is difficult to compare the prevalence of MetSyn in different populations, longitudinal comparisons can provide information for understanding the effects of driving forces, for setting intervention priorities and for evaluating community programs. In our study, we found that the crude prevalence of MetSyn increased sharply from 7.3% in 2002 to 24.5% in 2010, which means that 17.2% of normal adults developed MetSyn during an eight-year period [19]. One major factor driving this dramatic MetSyn escalation was likely the aging of the population as aging is closely related to MetSyn; the mean age increased from 44.9 in 2002 to 50.4 in 2010 [19]. The aging was more apparent in rural areas than in urban areas. In 2002, the urban residents were significantly older than the rural residents [19], but the difference narrowed in 2010 and was no longer statistically significant. The steeper aging trend in rural areas may be attributable to many young people migrating from rural areas to cities in search of a better life. This hypothesis is also in line with the higher increases we found in the prevalence of MetSyn and its components among rural residents than among urban residents.

Albeit that aging was a main driver of the dramatic escalation in MetSyn and vascular risk in this population, it should not be the greatest contributor. In fact, the prevalence of MetSyn and its components increased dramatically after controlling for age (Table 4). We speculated that rapid industrialization and urbanization may be the most important cause of the accelerating increase in MetSyn prevalence in this population. Between the 1940s and the late 1970s, the major challenge faced by Chinese authorities was to provide the people with sufficient food to meet their basic energy and nutritional requirements [33, 34]. However, since 1979, when China began to open up and reform its economy, it has enjoyed the most rapid economic development in the world. Guangdong is the first province where the Chinese leader Deng Xiao Ping started economic reforms and its GDP growth has been the fastest among all the provinces and autonomous regions of China during the past three decades. Statistics show that Guangdong’s GDP growth increased from RMB 410 in 1979 to RMB 44 736 in 2010 [18]. There were no data on food consumption for Survey 2010. However, the three nutrition surveys have also shown that the consumption of animal products increased three folds from 1982 to 2002 in Guangdong [18]. This rapid economic development and urbanization has increasingly promoted a sedentary lifestyle, an elevated consumption of energy-dense foods, and greater psychological stress, all of which increase the risk of vascular disease [11, 35].

Our results also show a greater increase from 2002 to 2010 in the prevalence of MetSyn and all its components (except hyperglycaemia) in rural residents than in urban residents. This means that the gap between urban and rural residents narrowed during the eight-year period. This result implies rapid economic development and urbanization had a greater impact on the prevalence of MetSyn and its components in rural residents, who generally have lower education levels and poorer access to health care than urban residents. This result is also in line with the Epidemiologic Transition theory, which predicts that in the early stages of a country’s economic development, chronic disease will generally be most apparent among the most educated and wealthiest members of the population; however, this trend will slow over time or even reverse as people realize the health hazards of a poor diet and poor lifestyle choices. In the mean time, the chronic disease burden shifts quickly to the people with lower socio-economic status.

Information on trends in MetSyn prevalence is relatively limited worldwide. Mozumdar *et al*. reported that the prevalence increased from 27.9% to 34.1% during a period of around a decade in U.S. adults [36]. Lim *et al*. reported that MetSyn prevalence in Korea increased from 24.9% to 31.3% between 1998 and 2007 in adults aged 20 years or over [37]. In comparison with the prevalence in 2002 [37], our results show a jump of 17.2% in MetSyn prevalence, as well as dramatic increases in its components, during an eight-year period in this population. Many previous studies show increase in the individual components and other vascular risk factors in other populations over the last several decades [38], but progress is much slower and the magnitudes are smaller. The more alarming results in this population are likely related to its much more rapid economic development and urbanization, which in turn has led to accelerating changes in nutrition, lifestyle, and socio-economic status. Previously published reports of our research also note a jump in diabetes and a slight increase in hypertension during the eight-year period [20, 21]. These findings suggest that the shifts in health and disease patterns accompanying an epidemiologic transition in populations with rapid economic development may be much faster than previously observed in Western countries [39]. The United Nations reported that around 5.7 billion (82.2%) of the world’s population live in less developed countries [40], where a booming economy is the top priority. Because the contemporary pace of industrialization and urbanization is much faster, the need to instigate comprehensive and rigorous prevention strategies for vascular disease in these developing countries is imperative.

## Conclusions

Our results show that cardiovascular risk escalated dramatically between 2002 and 2010 in this population experiencing the world’s fastest GDP growth in last three decades. The escalation may relate to the rapid economic development, which led to accelerating changes in nutrition, lifestyle, and socio-economic status. Our findings suggest that because the contemporary pace of industrialization and urbanization is much faster, the health transition nowadays among the developing second- and third-world countries may be much faster than what has been observed in Western countries.

## Authors’ information

Xiang Qian Lao and Wen Jun Ma are co-first authors.
